# Supplementary figures and images for: Artificial Intelligence for Prognosis of Gastro-Entero-Pancreatic Neuroendocrine Neoplasms
Source: Cancers (Basel). 2025 Jun 13;17(12):1981. doi: 10.3390/cancers17121981 (PMC12191315; doi:10.3390/cancers17121981)

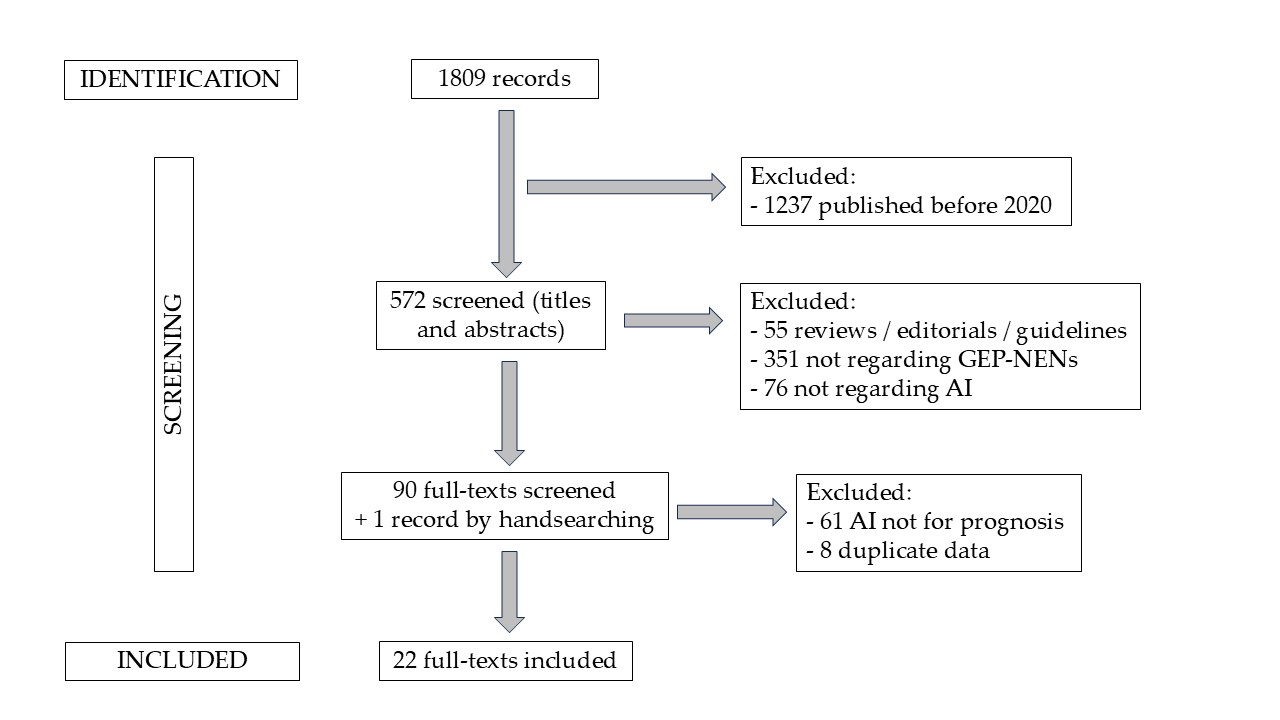

Supplement: Supplementary file 1 [file cancers-17-01981-s001.zip › cancers-3700535-supplementary.jpg]
